# Phylogeographic, morphometric and taxonomic re-evaluation of the river sardine, *Mesobola brevianalis* (Boulenger, 1908) (Teleostei, Cyprinidae, Chedrini)

**DOI:** 10.3897/zookeys.641.10434

**Published:** 2016-12-16

**Authors:** Megan A. Riddin, I. Roger Bills, Martin H. Villet

**Affiliations:** 1 Department of Zoology & Entomology, Rhodes University, African Street, PO Box 94, Grahamstown, 6140 South Africa; 2 South African Institute for Aquatic Biodiversity, Somerset Street, Grahamstown, 6140 South Africa

**Keywords:** Phylogeography, morphometrics, nomenclature, Mesobola, Engraulicypris, new species, new combinations, new synonym

## Abstract

The river sardine, *Mesobola brevianalis* (Boulenger, 1908), is the type species of *Mesobola* Howes, 1984. Standard phylogenetic analyses of partial sequences of the cytochrome oxidase I gene of individuals from populations across southern Africa that are currently identified as *Mesobola brevianalis* showed that these populations represent four genetically distinct allopatric lineages. Furthermore, *Engraulicypris sardella* (Günther, 1868), the type species of *Engraulicypris* Günther, 1894, was convincingly nested amongst these clades. These findings support synonymisation of *Engraulicypris* and *Mesobola* **syn. n.**; restoration of *Engraulicypris gariepinus* (Barnard, 1943), **stat. rev.** for the lower Orange River population; description of two new species, *Engraulicypris ngalala* **sp. n.** and *Engraulicypris howesi* **sp. n.** from the Rovuma and Kunene river systems, respectively; affirmation of the synonymy of *Engraulicypris brevianalis* (Boulenger, 1908), **comb. n.** sensu stricto and *Engraulicypris whitei* van der Horst, 1934; and restoration of *Engraulicypris bredoi* Poll, 1945, **stat. rev.** and *Engraulicypris spinifer* Bailey & Matthes, 1971, **stat. rev.** from *Mesobola*. Discriminant function analysis of a truss network of five traditional morphometric measurements and 21 morphometric measurements that characterised the shape of the fishes was used to seek morphological markers for the genetically distinct populations. Only *Engraulicypris gariepinus* was morphometrically distinctive, but live colouration differed between the lineages. Detailed taxonomic descriptions and an identification key for the species are provided.

## Introduction

The river sardine, *Mesobola brevianalis* (Boulenger, 1908), is a small, shoaling fish that favours the upper stratum of open waters particularly in rivers and dams in south central Africa (Engelbrecht and Mulder 1999), breeds in early summer, and feeds on planktonic crustaceans and insects (Hay et al. 2008). It is important for its potential as a food source for sympatric game and predatory fish, including indigenous nembwe (*Serranochromis robustus* (Günther, 1864)), silver catfish (*Schilbe intermedius* Rüppell, 1832) and tigerfish (*Hydrocynus vittatus* (Castelnau, 1861)), and introduced bass (*Micropterus* spp.) (Engelbrecht and Mulder 1999), and it is therefore used as bait by subsistence fisherman (Engelbrecht and Mulder 1999). However, little research has been done to inform the species’ management, perhaps due to its low commercial potential (Engelbrecht and Mulder 1999).

*Mesobola brevianalis* falls under several fisheries jurisdictions, occurring in a number of southern African river systems, including the Kunene, lower Orange, Okavango and Zambezi River systems (van der Horst 1934, Barnard 1943, Bell-Cross 1965, Skelton 2001, Hay et al. 2008, Ramollo 2011). It is considered alien to the Lower Zambezi River, since they were introduced into the Nyamombe River, a tributary of the Mazowe River in Mozambique (Kadye 2008). River sardines have also been found in the eastern coastal rivers from the Mfolozi and Mkhuze rivers (Skelton and Whitfield 1989, Hay et al. 2008) to the Limpopo River (Olivier et al. 2009) in South Africa, and in the Rovuma River system in Mozambique.

Populations from different river systems show subtle differences in morphology or colouration that may indicate cryptic species, but this potentially significant geographical variation in the river sardine is not reflected in its taxonomy. *Mesobola brevianalis* was described as *Neobola brevianalis* Boulenger, 1908 based on specimens from the Mkuzi River, South Africa. It was transferred to its current genus, *Mesobola* Howes, 1984, as the type species of that genus (Howes 1984, Eschmeyer et al. 2016). *Engraulicypris whitei* van der Horst, 1934 was described from the Aapies River, a tributary of the Limpopo system, and later synonymized with *Neobola brevianalis* by Jubb (1963: 15, 26, Eschmeyer et al. 2016). Subsequently, *Engraulicypris gariepinus* Barnard, 1943 was described from the lower Orange River and also synonymized with *Neobola brevianalis* by Jubb (1967: 42, Eschmeyer et al. 2016). The status of these taxa was not assessed using contemporary quantitative methods.

A morphometric and phylogeographic study was therefore undertaken to assess the taxonomy of the biogeographically distinct populations of *Mesobola brevianalis* *sensu lato* (i.e. including all taxonomic synonyms). Amongst other nomenclatural acts, the results support the synonymisation of *Engraulicypris* Günther, 1894 and *Mesobola* Howes, 1984, syn. n., the restoration of *Engraulicypris gariepinus* Barnard, 1943, stat. rev., and the description of two new species of *Engraulicypris*.

## Materials and methods

### Specimens

**Figure 1.**
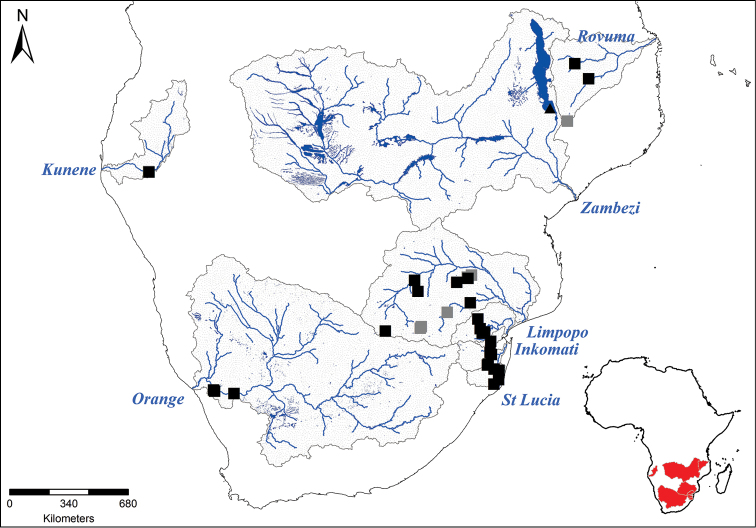
Map of the catchments and sampling sites in which the study species occur. p DNA and morphology: *Engraulicypris sardella*; n DNA and morphology: *Mesobola brevianalis* *s.l.*; n morphology only: *Mesobola brevianalis* *s.l.* (Generated by H. Retief, Rhodes University).

Specimens identified as *Mesobola brevianalis* (Boulenger, 1908) were collected from twelve river systems from ten African countries (Fig. 1, Tables 1, 2). The fish were collected under permit by various methods including hand, seine netting and electrofishing device. Specimens were killed by over-dosing in a mixture of clove oil and water and when possible, photographs were taken of the left side of the fish to record its live colouration. The specimens were then fixed in 10% formalin and specimens collected in the same event were placed together into a container with a waterproof label bearing the date, sample number, location, details of the capture and preservation methods, the sample and specimen numbers (Tables 1, 2). In the laboratory, the fixed specimens were transferred through a series of dilutions up into 70% ethanol for long-term preservation.

**Table 1.** Sample catalogue numbers and locality information for specimens from which DNA was extracted for phylogenetic analysis.

Taxon and Locality		Geocoordinates	SAIAB Catalogue Number	GenBank accession number
**OUTGROUPS**				
*Neobola bottegoi*				
	Wabe River	7.44°N 40.17°E		HM224178
*Chelaethiops congicus*				
	Malagarasi River	5.18°S 30.05°E	191919 DT10-A416	KX808580
*Raiamas salmolucius*				
	Lulua River			JX197004.1
*Opsaridium ubangiense*				
	Oubangui River	6.18°N 20.74°E		HM224193
*Engraulicypris sardella*				
	Lake Malawi			JX196997
		14.12°S 34.93°E	191026	HM418189
			191026 DT13-M066	KX788904
			191026 DT13-M038	KX788905
**INGROUP**				
*Mesobola brevianalis* *s.s.*				
	Albasini Dam	23.10°S 30.12°E	191910 RB12-Misc048	KX788875
			191910 RB12-Misc100	KX788876
	Olifants River	24.19°S 30.82°E	190710 RB13-B066	KX788873
			190710 RB13-B094	KX788874
	White Mbuluzi River	26.17°S 31.88°E	190657 RB13-B012	KX788888
			190657 RB13-B014	KX788889
	Crocodile River	25.53°S 31.33°E	190670 RB13-B040	KX788899
			190670 RB13-B050	KX788900
	Mlumati River	25.68°S 31.56°E	190621 RB13-B044	KX788896
			190621 RB13-B033	KX788897
		25.76°S 31.44°E	66145 S7	KX788898
	Sabie River	25.02°S 31.21°E	190665 RB13-B062	KX788895
	Usuthu River	26.86°S 31.91°E	66270	HM224176
			190635 RB13-B048	KX788883
	Pongolo River	27.40°S 31.70°E	190682 RB13-B269	KX788884
			190682 RB13-B252	KX788885
		27.35°S 31.75°E	188141 RB13-B279	KX788886
			188141 RB13-B262	KX788887
	Hluhluwe River	28.15°S 32.28°E	190719 RB13-B278	KX788880
			190719 RB13-B280	KX788881
			190719 RB13-B281	KX788882
	Mfolozi River	28.39°S 32.04°E	190676 RB13-B294	KX788890
			190676 RB13-B259	KX788891
	Mkhuze River	27.67°S 32.30°E	88674 PM09A211	KX788877
			88674 PM09A214	KX788878
		27.61°S 32.04°E	190643 RB13-B270	KX788879
*Mesobola whitei* topotypes				
	Limpopo River	23.00°S 27.94°E	101196 A	KX788892
			101196 B	KX788894
		25.65°S 26.43°E	187259 KW12-AT410	KX788893
*Mesobola gariepinus*				
	Orange River	28.87°S 18.61°E	78805 IRB-06-01	KX788901
		28.69°S 17.56°E	78822 IRB-06-03	KX788902
		28.75°S 17.61°E	78831 IRB-06-04	KX788903
*Mesobola howesi* sp. n.				
	Kunene River	17.41°S 14.22°E	78759 A ES06_A_54	KX788912
			78759 B ES06_A_54	KX788913
*Mesobola ngalala* sp. n.				
	Lake Chiuta	14.91°S 36.02°E	191029 DT13-M100	KX788906
	Lucheringo River	11.82°S 36.22°E	74087 A N39	KX788909
			74087 B N39	KX788910
	Rovuma River	12.60°S 36.94°E	73944 A N22	KX788907
			73944 B N22	KX788908
			73944 C N22	KX788911

**Table 2.** Sample catalogue numbers and locality information for specimens from which measurements were taken for morphometric analysis.

Species	Locality	Geocoordinates	SAIAB Catalogue number	Number of specimens
*Engraulicypris sardella*	Lake Malawi	14.12°S 34.93°E	191026	5
*Mesobola brevianalis*	Albasini Dam	23.10°S 30.12°E	191910	20
	Luvhuvhu River	22.90°S 30.70°E	82589	7
	Limpopo River	22.99°S 27.94°E	RB13-Limpopo1	9
	Mbwedi River	22.84°S 30.66°E	53570	4
	Mutshindudi River	22.86°S 30.69°E	53561	2
	Olifants River	24.67°S 29.62°E	61119	10
	–	24.18°S 30.82°E	RB13-Mes26	31
	White Mbuluzi River	26.16°S 31.87°E	RB13-Mes19	32
	Crocodile River	25.52°S 31.32°E	RB13-Mes22	33
	Mlumati River	25.68°S 31.56°E	RB13-Mes21	32
	Nkomati River	25.76°S 31.44°E	66145	18
	Sabie River	25.02°S 31.20°E	RB13-Mes23	15
	Mtindzekwa River	26.74°S 31.83°E	RB13-Mes23	31
	Usuthu River	26.86°S 31.91°E	66270	18
	Hluhluwe River	28.38°S 32.28°E	RB13-Mes04	39
	Mfolozi River	28.38°S 32.03°E	RB13-Mes02	5
	Mkhuze River	27.59°S 32.41°E	RB13-Mes05	60
*Mesobola whitei* syntypes	Aapies River	25.42°S 28.28°E	30041	9
*Mesobola gariepinus*	Orange River	28.69°S 17.56°E	78805	16
*Mesobola howesi*	Kunene River	17.41°S 14.22°E	78759	6
*Mesobola ngalala*	Lucheringo River	11.82°S 36.22°E	74087	4
	Rovuma River	12.60°S 36.94°E	73944	25
	Lake Chiuta	14.78°S 35.83°E	–	28
**Total**:				**461**

When a fresh or ethanol-preserved fish was selected for genetic analysis, the entire caudal fin, or a muscle tissue sample taken between the end point of its dorsal fin and the beginning of its caudal fin, was placed in 95% ethanol in a separate microcentrifuge tube. The tissue samples and the whole specimens were catalogued into the South African Institute for Aquatic Biodiversity (SAIAB), Grahamstown.

### Phylogenetic relationships

The relationships of the sampled populations identified as *Mesobola brevianalis* and representatives of its near relatives in the Chedrini (Tang et al. 2010; Liao et al. 2012) were estimated using phylogenetic analysis of mtDNA sequences. Each tissue sample used for DNA extraction (Table 1) was dried completely before being placed in a new microcentrifuge tube. DNA was extracted using the DNeasy® blood and tissue kit (Qiagen, Valencia, CA) and the NucleoSpin® Tissue kit (Machery-Nagel GmbH & Co. KG) following the manufacturer’s instructions for animal tissue isolation, except that the incubation period was 12 h to allow for complete tissue digestion and the final dilution step was performed with 50 µl (rather than 200 µl) nuclease-free distilled water during extraction with the DNeasy® kit to provide a higher concentration of DNA. The concentration and purity of each DNA extract was determined by using a NanoDrop 2000 Spectrophotometer. The DNA concentration, A260, A280, 260/280 and 260/230 values were documented to ensure that the DNA was sufficiently concentrated and pure.

A 658 basepair (bp) fragment of the protein-coding *Cytochrome Oxidase 1* (COI) mitochondrial gene was amplified using the LCOI490 and HCO2198 primer set (Folmer et al. 1994). The PCR conditions for this gene fragment were 94°C for 1 min, 45°C for 1.5 min, 72°C for 1.5 min, annealing of 94°C for 1 min, 50°C for 1.5 min and 72°C for 1 min for 40 cycles and a final elongation stage at 72°C for 5 min. The PCR products was electrophoretically separated on a 1% agarose gel at 80 V for 30 min. Attempts to amplify the protein-coding *Recombination Activating Gene 1* (RAG1) nuclear gene failed, and although the 28S rRNA nuclear gene was amplified, it (predictably) showed no informative variation within *Mesobola*.

Sequencing by capillary electrophoresis was conducted by Macrogen Inc. (Seoul, South Korea) using the amplification primers. The forward and reverse nucleotide sequences were aligned using the ClustalX multiple sequence alignment module (Larkin et al. 2007) within the BioEdit sequence alignment software (Hall 2004) to form consensus sequences and deposited in GenBank (https://www.ncbi.nlm.nih.gov/genbank) (Table 1).

The sister group to *Mesobola* is contentious (Howes 1980, 1984, Tang et al. 2010; Liao et al. 2012), so representative species of several genera, including *Chelaethiops* Boulenger, 1899, *Engraulicypris* Günther, 1894, *Neobola* Vinciguerra, 1895, *Opsaridium* Peters, 1854 and *Raiamas* Jordan, 1919, were chosen as outgroup taxa to root the phylogenetic analysis. The relevant additional sequences were either generated from tissue samples or downloaded from GenBank (https://www.ncbi.nlm.nih.gov/genbank) (Table 1).

All of the sequences were aligned using ClustalX (Larkin et al. 2007) and saved in a Nexus-format file. MrModelTest (Nylander 2004) was used to access the model of best fit for the sequences using the Akaike Information Criterion (Akaike 1973), and the TrN+I+G model was selected and used to build a Bayesian inference tree in MrBayes (Huelsenbeck and Ronquist 2001) using a total of ten million generations (until the split frequency was below 0.05), with a tree sampled every 1000 generations. After examining the trace file, the first 20% of the sampled trees were discarded as burn-in. The Bayesian inference trees were viewed and annotated using TreeView (Page 1996).

### Morphological characterization

The morphology and live colouration of representatives of each clade was examined in details for diagnostic traits; measures follow Howes (1980, 1984). Preserved specimens were each placed into a black- or white-based container (to provide contrast) filled with 70% ethanol and a photograph was taken of its left side using a Canon 550D SLR camera (18.1 megapixels) and 50 mm fixed macro lens. A scale bar was included in each photograph to calibrate measurements. Each specimen was then labelled with waterproof paper bearing its specimen number and photograph number, placed in a separate vial for further reference, and returned to its collecting lot.

The available type specimens of *Mesobola brevianalis* and its synonyms, and of *Engraulicypris sardella* were also examined using photographs supplied by the Natural History Museum, London (BMNH).

**Figure 2.**
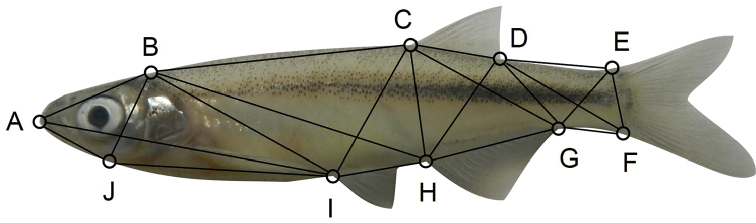
Truss network used for morphometric analysis, defined by ten landmarks **A–J**.

Based on these results, morphometric analysis of selected specimens (Table 2) from each major clade found in the phylogenetic analysis was used to find morphological features suitable for identification. The photographs were imported into the imaging software, AnalySIS Docu (Olympus Soft Imaging Systems: http://www.soft-imaging.net/) to measure six standard linear measurements: standard length (SL), orbit length, snout-to-orbit distance, and the lengths of the dorsal, anal and pelvic fins. A box truss network (Strauss and Bookstein 1982) of 21 measurements was used to capture the shape of each fish, based on ten landmark points (Fig. 2) that lay in areas of strong skeletal support, where distortion of soft tissue was likely to be minimal. All measurements were entered into a spreadsheet with each specimen’s collection number, geographical origin (country, river, river system) and nomenclatural status (e.g. holotype, syntype). Measuring and transcription errors were sought using scatter plots and corrected.

The measurement data were log-transformed to rectilinearise allometric variation (Strauss and Bookstein 1982), and a principal component analysis was used to seek morphological groups in the samples. A discriminant function analysis was performed to pinpoint diagnostic measurements of taxa defined by the genetic analysis. Both analyses were done using the Statistica 12 (http://www.statsoft.com/Products/STATISTICA-Features/Version-12) software package.

### Taxonomy

Type specimens and their metadata were housed in the South African Institute for Aquatic Biodiversity, Grahamstown (SAIAB), the Albany Museum, Grahamstown (AMGT) and the Natural History Museum (BMNH), London. Photographs of the holotypes of *Mesobola brevianalis* and *Engraulicypris sardella* were received from the BMNH as the specimens were too fragile to transport. Catalogued SAIAB specimens of undescribed species was selected for description based on their physical condition (e.g. fin rays and scales intact) and whether they had associated genetic sequences.

Specimens were photographed with a scale bar. Measurements were made on each specimen with standard unbranded electronic digital callipers. The holotype photographs were measured using AnalySIS Docu software, but measurements that involved the width of the specimen including body width or inter-orbit length could not be measured or included in the description.

Meristic data, including fin ray counts, where gathered using a Leica Zoom 2000 microscope. Scale counts were made on a maximum of only three specimens because it required dyeing specimens with Alizarin Red for an average of five-to-ten minutes and then placing them directly into Acid Blue dye for a further five-to-ten minutes, after which visualising the scales was still very difficult. Because the dye did not wash out well, scale counts were not be made on type specimens. Vertebra counts were made on X-rays of some specimens including all holotypes except for the holotype of *Engraulicypris sardella* for which no X-ray was available. A single specimen from each population was cleared and stained using standard methods (Taylor and van Dyke 1985), preserved in 70% glycerol, and dissected to count the gill rakers on both the ceratobranchial and epibranchial of the first gill arch.

The data were used to populate a character database in the DELTA software package (Dallwitz 1980, Dallwitz et al. 1993), which was used to generate the species descriptions and key.

## Results and discussion

### Phylogenetic relationships

**Figure 3.**
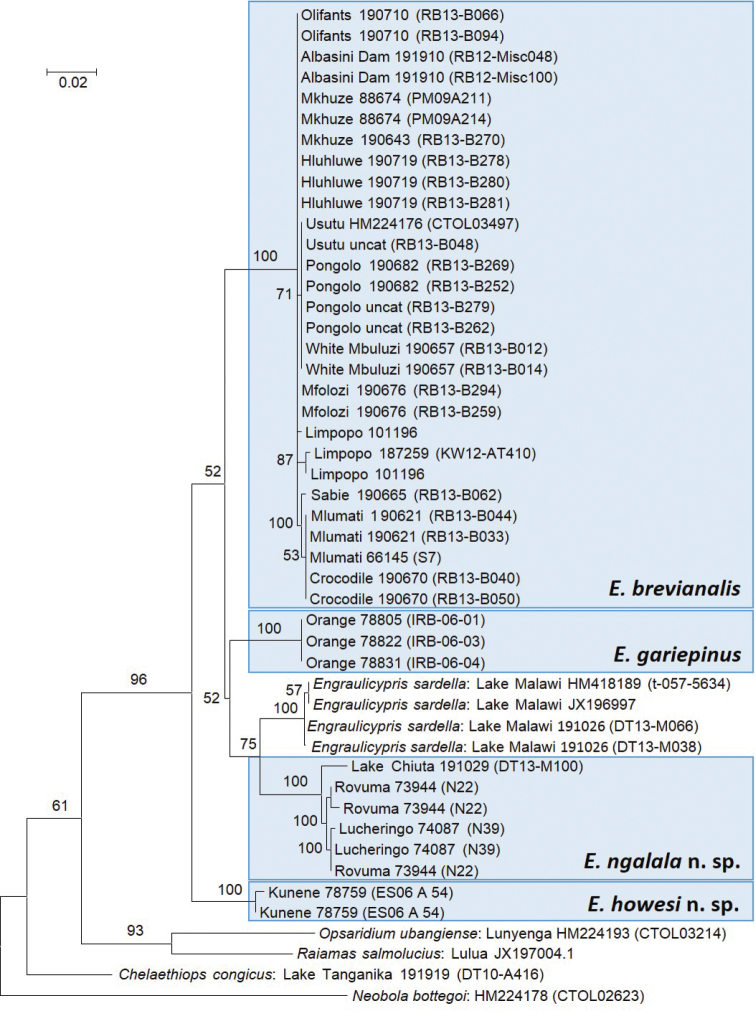
Maximum-likelihood phylogram based on partial sequences of the COI gene. Bootstrap support values were attained using a heuristic tree search and 1000 replicates. Numbers following locality names represent SAIAB catalogue numbers and GenBank accession numbers (in brackets). The shaded boxes enclose well-supported clades that were identified as populations of *Mesobola brevianalis* in the past. The scale bar represents the number of base substitutions per site.

The Bayesian phylogenetic analysis with a maximum-likelihood model showed that the biogeographically disparate populations identified as *Mesobola brevianalis* represent independent evolutionary clades (support values = 100% in all cases) with relative branch lengths (i.e. numbers of base substitutions per site) indicating larger average evolutionary divergence between the clades than within them (Fig. 3). These clades were collectively paraphyletic with respect to *Engraulicypris sardella* (Fig. 3), but the monophyly of the whole ensemble received bootstrap support of 96%.

Support for relationships between the independent clades was weak, possibly suggesting a relatively rapid radiation, with the strongest evidence (p = 0.755) supporting a biogeographically plausible sister-group relationship between *Engraulicypris sardella* from Lake Malawi and the population from the neighbouring Rovuma River system (Fig. 3). The Malawi Rift Basin began to form ~8.6 mya, in the Late Miocene (Delvaux 1995; Danley et al. 2012), cutting across the headwaters of the Palaeo-Rovuma River. This would provide a first approximation for the time of vicariance of these two clades.

The sister group to *Mesobola* remains uncertain for the same reasons that affected the study by Tang et al. (2010), which used four genes and many more taxa: limited taxon sampling within the African radiation of Chedrini and the involvement genera like *Raiamas* and *Opsaridium* that are potentially polyphyletic and not represented by their type species. The average evolutionary divergence between taxa is represented as number of base substitutions per site (Fig. 3).

### Morphological identification

**Figure 4.**
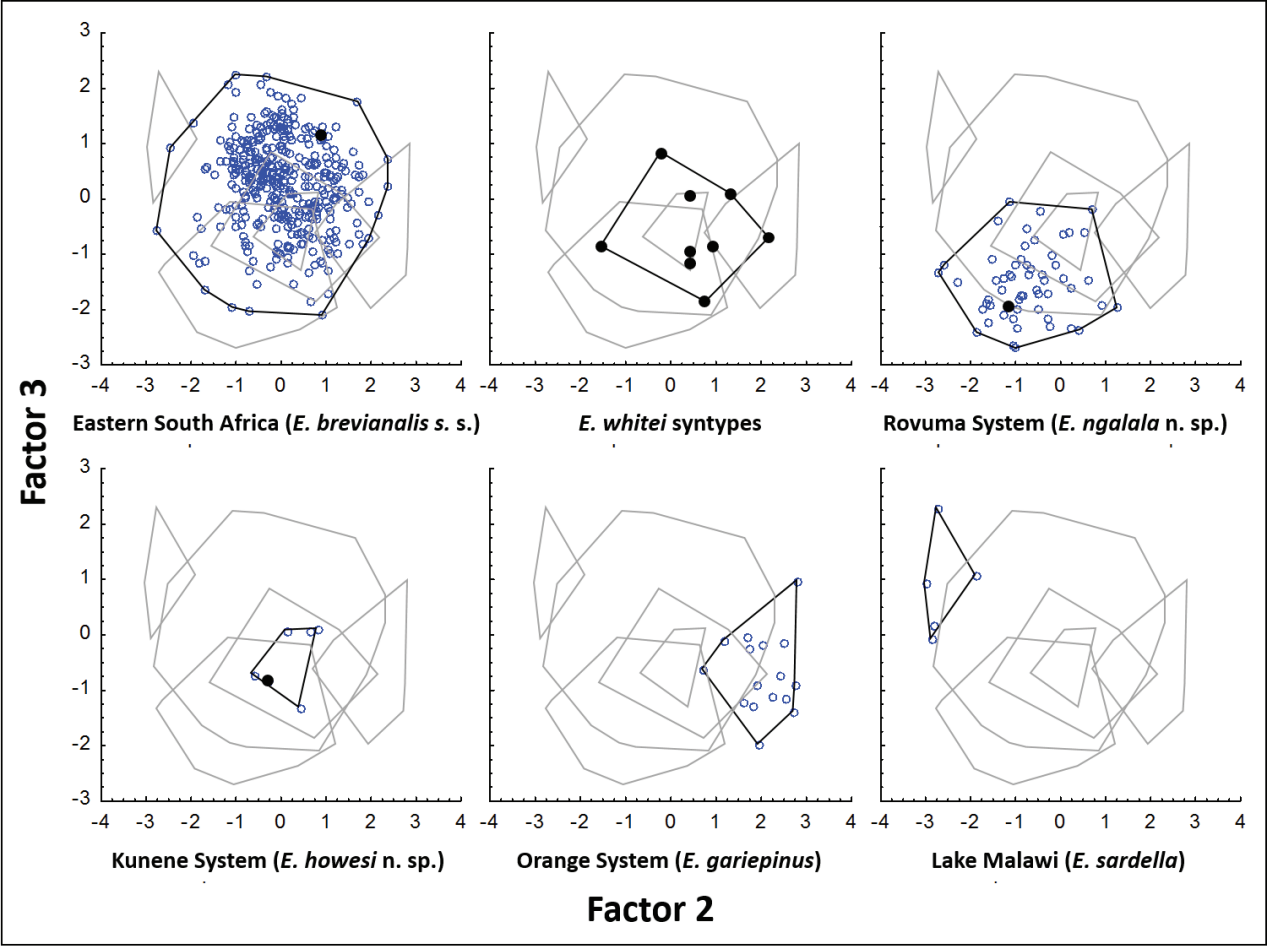
Plots of the second and third canonical axes of a principle component analysis of traditional and truss-based morphometric measurements of representative specimens of *Engraulicypris sardella* and various populations referred to *Mesobola*, separated by taxon. Type specimens are marked with solid circles.

Although the phylogenetic analysis showed distinct populations within *Mesobola brevianalis* *sensu lato*, these could not be detected in a principal component analysis of the morphometric data. The first Eigenvector summarised 89% of the variance and its coefficients were all fairly similar in magnitude and uniform sign (Table 2), indicating that it summarised a general effect in the data, i.e. size, as is usual with morphometric analyses of organisms when variation in the sizes of specimens outweighs their variation in shape. Being orthogonal to the first axis, the remaining axes summarised variation in shape and allometry independent of gross differences in size. A plot of the second and third axes (Fig. 4) showed that populations from the Kunene River and eastern South Africa (including the syntypes of *Engraulicypris whitei*) overlapped entirely in that morphospace, and partially overlapped those of the Rovuma and Orange rivers, which were mutually distinct. This supported the synonymization of *Mesobola brevianalis* and *Engraulicypris whitei*, which both occupy the Limpopo River system, and explains why most of the populations have not yet been recognised as distinct taxa. The second axis summarised 2.6% of the variance and differentiated the Rovuma and Orange River populations by emphasising truss measurements DE, DF, CD, DG and dorsal fin length (Table 3), which described the shapes of the caudal peduncle and the dorsal fin (Fig. 2). The third axis summarised 2.2% of the variance in morphology and emphasised eye length and the truss measurements AB, AJ and BJ (Table 3), which all described the head (Fig. 2), but did little to separate the populations further (Fig. 4). The remaining 24 axes collectively summarised only 6.1% of the variation and did not describe patterns that related to the populations.

**Table 3.** First three Eigenvectors of a principle component analysis of the morphometric data. Coefficients in bold lie outside the 95% confidence interval for the mean coefficient of each axis, and are therefore unusually influential in dispersing specimens on that axis.

Measurement	PCA			DFA		
	Factor 1	Factor 2	Factor 3	Root 1	Root 2	Root 3
**A–B**	-0.185	-0.158	**0.424**	0.101	-0.104	**0.470**
**A–I**	-0.199	0.002	0.171	0.041	0.012	0.315
**A–J**	-0.189	0.126	0.253	0.119	0.083	0.332
**B–C**	-0.196	0.043	-0.310	-0.070	0.094	0.291
**B–H**	-0.197	0.109	-0.154	0.001	0.100	0.310
**B–I**	-0.197	0.110	0.165	0.116	0.064	0.288
**B–J**	-0.189	-0.133	0.357	0.075	-0.056	0.419
**C–D**	-0.187	0.357	0.067	0.127	0.093	0.338
**C–G**	-0.197	0.183	0.018	0.100	0.090	0.303
**C–H**	-0.199	0.063	-0.148	0.019	0.098	0.286
**C–I**	-0.200	0.008	-0.169	-0.024	0.081	0.349
**D–E**	-**0.174**	-**0.570**	-0.094	-0.088	-**0.136**	0.273
**D–F**	-0.187	-**0.420**	-0.064	-0.045	-0.103	0.284
**D–G**	-0.196	0.287	-0.012	0.087	0.126	0.296
**D–H**	-0.199	0.129	-0.115	0.023	0.095	0.299
**E–F**	-0.198	0.084	0.038	0.076	-0.016	0.281
**E–G**	-0.201	-0.046	-0.074	0.004	0.038	0.295
**F–G**	-0.199	-0.072	-0.089	-0.009	0.037	0.276
**G–H**	-0.190	-0.138	-0.216	-0.067	0.026	0.272
**H–I**	-0.192	0.027	-0.126	-0.016	0.088	0.404
**I–J**	-0.189	-0.113	0.066	-0.055	-0.042	0.256
**Caudal fin length**	-0.193	0.006	-0.067	0.012	0.003	0.285
**Caudal length**	-0.202	-0.035	-0.103	-0.026	0.041	0.350
**Dorsal fin length**	-0.185	0.264	-0.168	0.005	0.107	0.259
**Eye length**	-0.187	-0.033	**0.413**	0.152	-0.071	0.295
**Pelvic fin length**	-0.186	-0.177	-0.198	-0.076	0.031	0.275
**Snout-to-eye distance**	-0.182	0.016	0.188	-0.002	0.020	0.409
**Eigenvalue**	24.070	0.695	0.595	2.557	1.412	0.810

**Figure 5.**
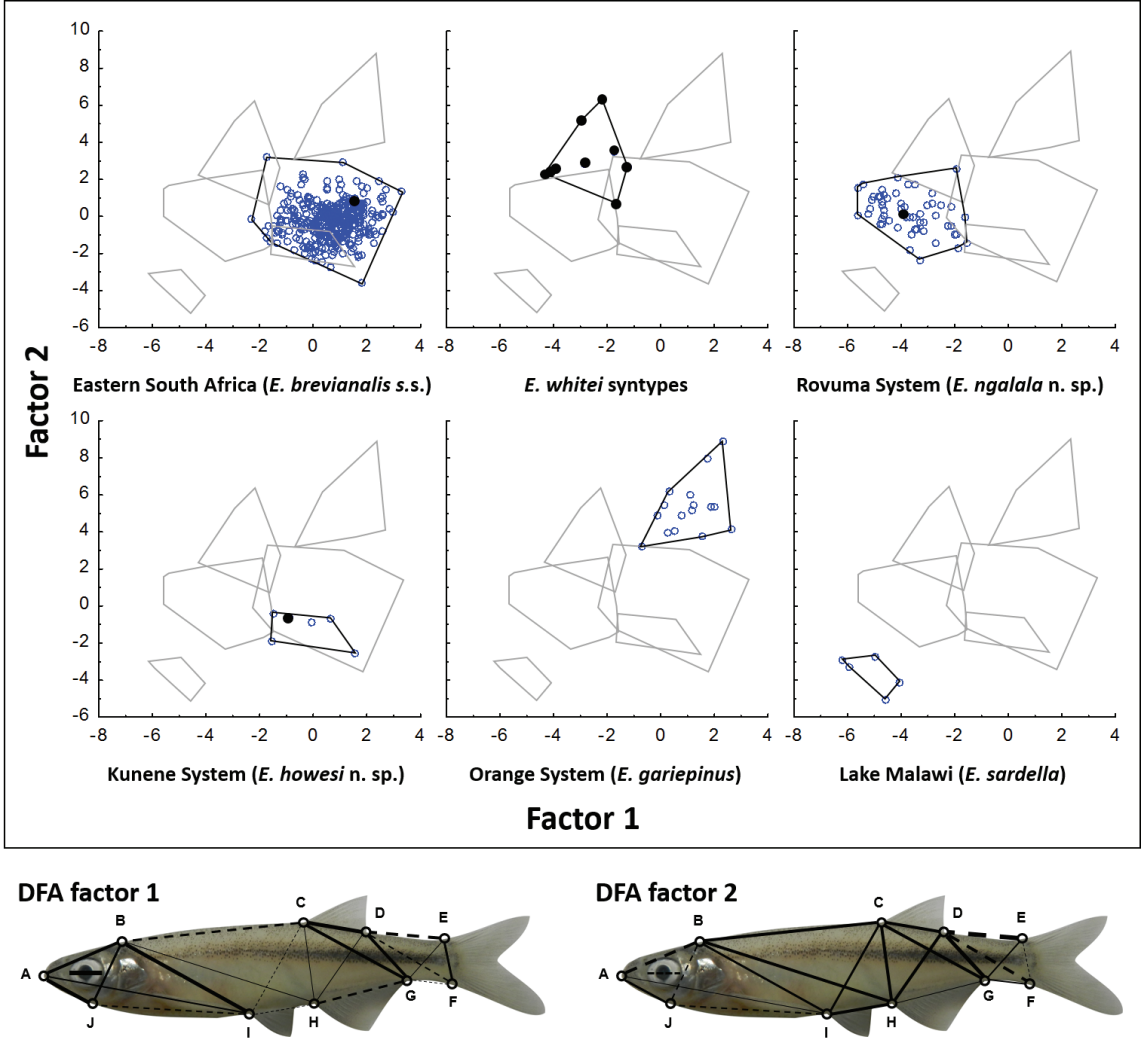
Plots of the first two canonical axes of a discriminant function analysis of traditional and truss-based morphometric measurements of representative specimens of *Engraulicypris sardella* and various populations referred to *Mesobola*, separated by taxon. Type specimens are marked with a solid circle.

Discriminant function analysis of the morphology of the genetically well-supported *Mesobola* populations and *Engraulicypris sardella* successful assigned most specimens to their population of origin (Table 4; Fig. 5), although *Mesobola brevianalis* *sensu stricto* and *Engraulicypris howesi* overlapped substantially in morphospace, at least on the first two canonical axes (Fig. 5). The first canonical axis tended to have negative weights for measurements along the body axis and positive weights for those across the body axis (Table 3; Fig. 5), thus describing the elongation of the body. The second axis contrasted measurements involving the dorsal fin with those of the caudal peduncle (Table 3; Fig. 5), while the third axis did showed no clear morphological pattern in its weights (Table 3).

**Table 4.** Classification matrix resulting from a discriminant function analysis of the morphometric data. Cells in bold contain misclassifications.

True identity	Predicted classifications						Percent correct
	*sardella*	*brevianalis*	*whitei*	*gariepinus*	*ngalala*	*howesi*	
*sardella*	5	0	0	0	0	0	100.0
*brevianalis*	0	365	**1**	0	**2**	0	99.2
*whitei*	0	**1**	8	0	0	0	88.9
*gariepinus*	0	0	0	16	0	0	100.0
*ngalala*	0	**7**	0	0	50	0	87.7
*howesi*	0	0	0	0	0	6	100.0
Total	5	373	9	16	52	6	97.6

**Table 5.** Morphometric measurements and meristic counts for *Engraulicypris brevianalis*.

Measurement	n	Holotype	Max	Min	Mean	SD
SL	6	67.39	67.39	41.13	46.46	10.30
Head length (%SL)	6	16.56	24.57	19.74	21.20	1.86
Head depth (%HL)	6	11.59	86.24	69.80	74.61	6.09
Snout length (%HL)	6	3.53	37.96	21.29	32.95	5.96
Orbit diameter (%HL)	6	5.65	45.85	34.14	39.69	4.68
Postorbit length (%HL)	6	6.34	38.27	25.62	30.68	4.74
Inter-orbit length (%HL)	5	Unknown	47.19	36.40	44.52	4.59
Predorsal length (%SL)	6	44.35	65.80	62.36	64.00	1.33
Prepelvic length (%SL)	6	33.46	50.78	46.48	48.23	1.80
Dorsal fin Length (%SL)	6	12.32	18.64	16.01	17.55	0.97
Pectoral fin length (%SL)	6	13.40	22.32	19.88	21.66	0.99
Pelvic fin length (%SL)	6	8.98	13.66	11.37	12.31	0.97
Anal fin length (%SL)	6	12.08	17.92	14.33	16.07	1.33
Body depth (%SL)	6	14.27	23.54	20.53	21.96	1.10
Body width (%SL)	5	Unknown	13.46	10.69	12.08	1.05
Caudal peduncle length (%SL)	6	10.04	18.10	14.10	15.74	1.57
Caudal peduncle depth (%SL)	6	6.78	11.01	9.52	10.24	0.58
Meristics	n	Holotype	Range			
Dorsal-fin rays	5	Unknown	ii+8 (n = 5)			
Anal-fin rays	5	Unknown	iii+13 (n = 1), iii+14 (n = 3), iii+15 (n = 1)			
Pectoral-fin rays	5	Unknown	i+10 (n = 4), i+11 (n = 1)			
Pelvic-fin rays	5	Unknown	i+7 (n = 5)			
Lateral line scales	2	Unknown	53 (n = 1), 57 (n = 1)			
Caudal peduncle scale	2	Unknown	18 (n = 2)			
Scale rows lat. line-dorsal	2	Unknown	9 (n = 1), 11 (n = 1)			
Scale rows lat. line-pelvic	2	Unknown	2 (n = 2)			
Scale rows lat. line-anal	2	Unknown	2 (n = 2)			
Total vertebrae	5	37	37 (n = 1), 38 (n = 4)			
Abdominal vertebrae	5	19	18 (n = 2), 19 (n = 3)			
Caudal vertebrae	5	18	19 (n = 4), 20 (n = 1)			
Rib pairs	5	14	13 (n = 1), 14 (n = 3), 15 (n = 1)			

## Taxonomy

Because *Engraulicypris pinguis* Günther, 1894 (= *Engraulicypris sardella* (Günther, 1868): Lévêque and Daget 1984, Eschmeyer et al. 2016) is the type species of *Engraulicypris* by monotypy, we resolve the genus-level paraphyly evident in the phylogeographical analysis (Fig. 3) by synonymising *Engraulicypris* Günther, 1894 and *Mesobola* Howes, 1984, syn. n. and transferring *Mesobola brevianalis* to *Engraulicypris brevianalis* (Boulenger, 1908), comb. n. We also restore two other species currently placed in *Mesobola* but originally placed in *Engraulicypris* by their authors (Eschmeyer et al. 2016): *Engraulicypris bredoi* Poll, 1945, stat. rev. and *Engraulicypris spinifer* Bailey & Matthes, 1971, stat. rev.

The species-level paraphyly in the phylogeographical analysis (Fig. 3) can be resolved by recognising the independent populations as species. In South Africa, specimens from the eastern populations of *Mesobola* grouped with specimens from the type locality of *Engraulicypris brevianalis* (Fig. 3) and were somewhat phylogenetically intermingled with specimens from the western populations from which *Mesobola whitei* was collected. These two species are therefore either synonymous or show incomplete lineage sorting or hybridization. The lower Orange River population can be recognised by restoring *Engraulicypris gariepinus* stat. rev. from synonymy with *Mesobola brevianalis*. *Engraulicypris bredoi* and *Engraulicypris spinifer* occur in Lake Albert and the Malagarasi River system, respectively (Lévêque et al. 1991), and are therefore unlikely to represent the Kunene and Rovuma River populations, for which there are thus currently no names available.

### Descriptions

#### Engraulicypris

Taxon classificationAnimaliaCypriniformesCyprinidae

Günther, 1894

Engraulicypris Günther, 1894: 626 (type species: Engraulicypris pinguis Günther, 1894 (= Barilius sardella Günther, 1868: Lévêque and Daget 1984, Eschmeyer et al. 2016))
= Mesobola Howes, 1984: 168 syn. n. (type species: Neobola brevianalis Boulenger, 1908)

##### Diagnosis.

With the synonymisation of *Mesobola* and *Engraulicypris*, Günther’s (1894) diagnosis of *Engraulicypris* must be modified to include the species assigned to *Mesobola*. *Engraulicypris* is a genus of moderately small African chedrin barbs (*sensu* Tang et al. 2010; Liao et al. 2011, 2012) identified by a lack of a scaly lobe at the base of the pelvic or pectoral fin; a large mouth reaching the anterior border of the orbit or beyond; a dorsal fin origin originating behind midpoint of standard length, more or less above the origin of the anal fin; a pectoral fin not reaching the origin of the anal fin; and body colouration lacking vertical bars or bands. Osteological characters are discussed by Liao et al. (2011, 2012) for *Mesobola* and by Liao et al. (2012) for *Engraulicypris*.

##### Live colouration.

**Figure 6.**
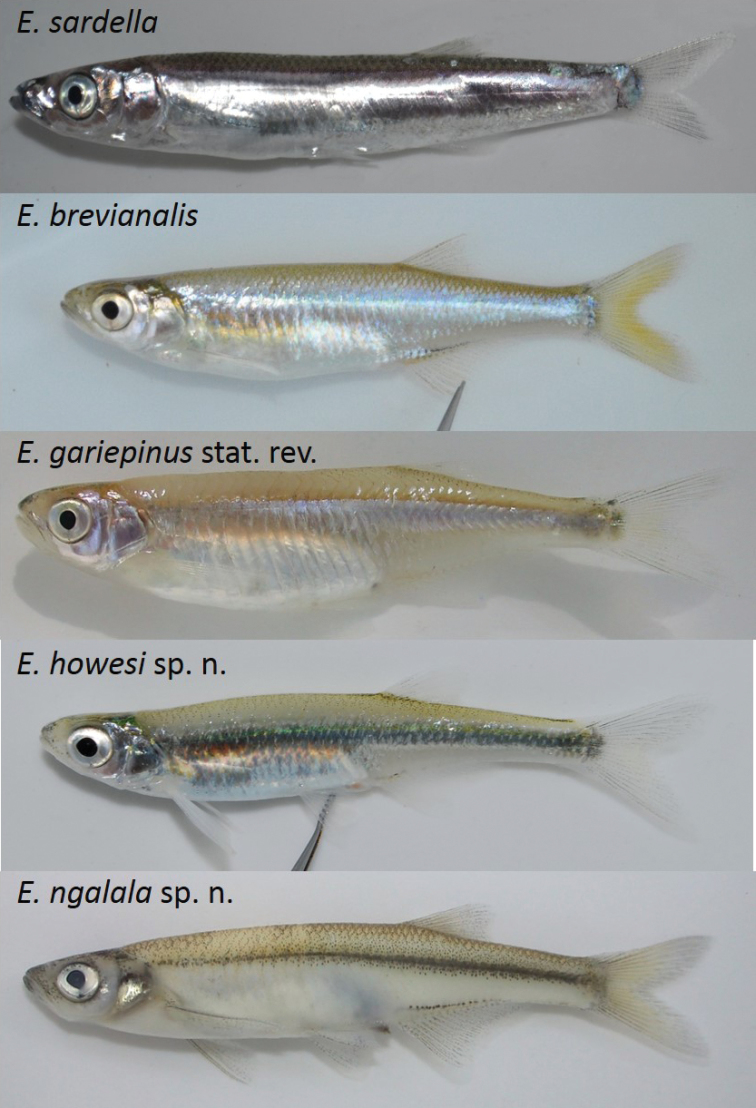
Photographs of fresh specimens of *Engraulicypris* species.

(Fig. 6). Body without vertical bars or bands.

##### Etymology.

*Engraulicypris* alludes to the anchovy-like form (eggraulis, -eos [eggraulis, -eos]; Greek) of these relatives of the carp (kyprinos [kyprinos]; Greek).

##### Distribution.

Southern and Eastern Africa.

#### Engraulicypris brevianalis

Taxon classificationAnimaliaCypriniformesCyprinidae

(Boulenger, 1908) comb. n.

Neobola brevianalis Boulenger, 1908. *Annals of the Natal Government Museum* 1(3): 281. Holotype: unsexed; “Mkuzi River, Zululand, Transvaal [sic]” [BMNH 1907.4.17.90] in formalin [BMNH].
= Engraulicypris whitei van der Horst, 1934. *Annals of the Transvaal Museum* 15(3): 281, unnumbered fig. Syntypes: 5 unsexed, Petronella [SAIAB 30040 ex TMP 15024]; 4 unsexed, Hammanskraal [SAIAB 30041 ex TMP 16022] in formalin [SAIAB].

##### Material examined.

***Engraulicypris brevianalis*: Holotype**, BMNH No 1907.4.17: 90, SL 67 mm. “Mkuzi River, Zululand, Transvaal”. [BMNH]. ***Engraulicypris whitei*: Syntypes**, SAIAB 30040 (ex TM 15024) (5) and SAIAB 30041 (ex TM 16022) (4), “Aapies River (Limpopo System) near Petronella and near Hammanskraal (Transvaal)”. Other material, see Table 2.

##### Diagnosis.

Caudal fin membrane clear towards vivid yellow at fork; anal fin extending two thirds of length of caudal peduncle; caudal peduncle moderately long; operculum entirely (not partially) shiny; body midline silver (not black); iris dark to light grey (not white); head with tubercles along lower jaw and lower head in breeding males; snout rounded (not pointed), darker dorsally; pelvic fin melanophores absent.

##### Morphology.

**Figure 7.**
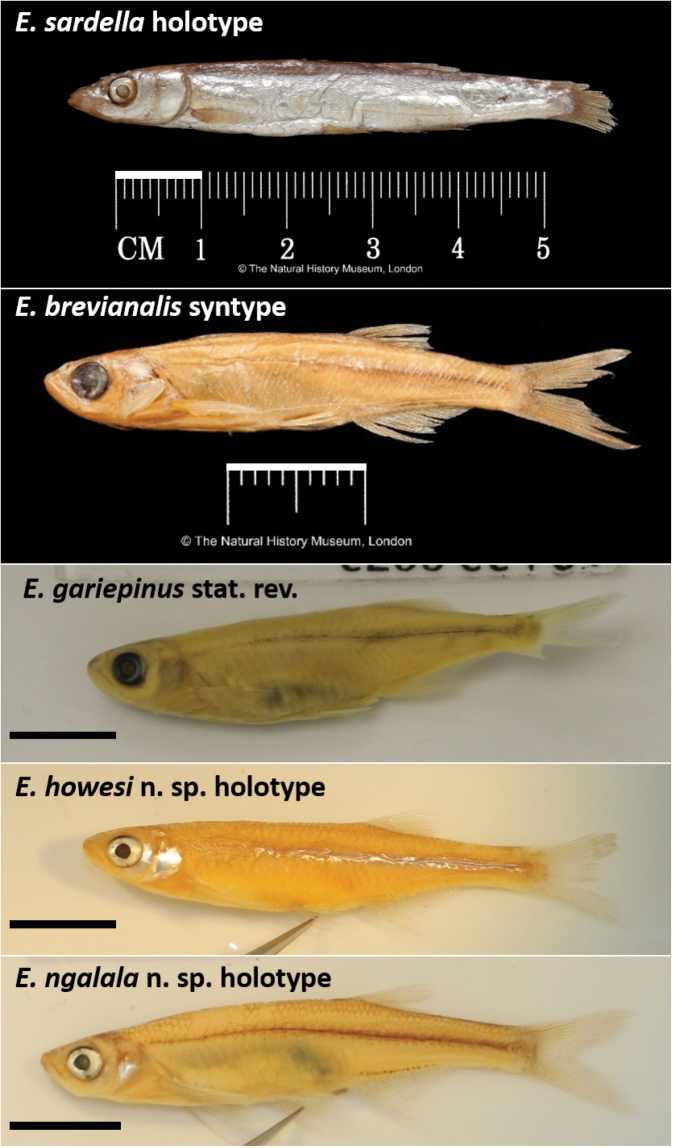
Photographs of preserved type specimens of *Engraulicypris* species. Scale bars = 1 cm.

**Figure 8.**
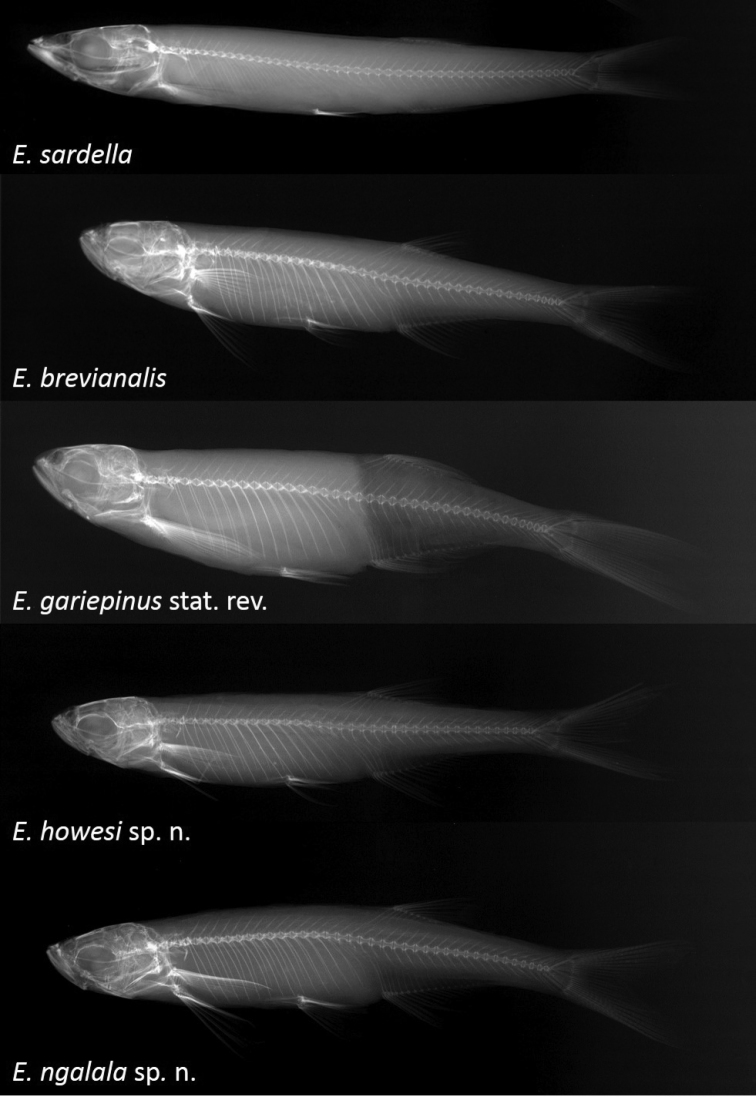
X-ray images of specimens of *Engraulicypris* species.

(Figs 6–8; Table 5). Maximum SL 75 mm. Body elongated; somewhat fusiform; laterally compressed. Maximum body depth at middle pelvic and pectoral fin origin. Pre-dorsal profile straight or slightly convex behind head. Head length 20% SL; with tubercles along lower jaw and lower head. Snout rounded; short; 30% of head length. Mouth terminal; slightly crescent-shaped with long anterior side; reaching anterior border of orbit. Nostrils large; level with dorsal margin of eye; separated from orbit by less than one orbit radius. Tubular anterior naris short; adjacent to open posterior naris. Eye lateral; visible from above and below (more prominent); diameter 35% of head length. First gill arch with 8+3 gill rakers on cerato- and epibranchial arms, respectively. Gill rakers long; pointed; widely-spaced. Pharyngeal bones in three rows. Pharyngeal teeth 4,3,2–2,3,4; robust and long; falcate.

Modal fin formulae in Table 5. Fins large in relation to body size. Dorsal fin closer to caudal fin than tip of snout; more or less above origin of anal fin; length 17% SL; posterior margin straight; rays soft; anterior-most branched fin ray longest. Pectoral fins largest; reaching ^1^/_2_ to ^3^/_4_ distance to base of pelvic fin; fin lacking lobe at base. Pelvic fins reaching ^2^/_3_ distance to base of anal fin; relatively small; pointed; fin lacking a basal lobe. Anal fin moderately long; extending ^2^/_3_ length of caudal peduncle; last unbranched ray longest. Ano-genital opening at anterior of base of anal fin. Caudal peduncle moderately long. Caudal fin forked; lobes with slightly concave interior and extending into point; upper lobe shorter.

Scales small to medium relative to body size; in regular rows; cycloid, slightly elongate; radially striate. Base of anal fin lacking sheath scales. Lateral line present; complete; dipping sharply towards ventral at tip of pectoral fin; joining midline at posterior of caudal peduncle; scale count 53–57 (n = 2) along lateral line, 18 around caudal peduncle.

##### Live colouration.

(Fig. 6). Body silver, without vertical bars or bands. Dorsum pale brown with small dark brown melanophores, midline silver. Snout darker dorsally. Operculum entirely metallic silver. Iris dark to light grey. Dorsal fin membrane clear; rays clear with olive melanophores; fading towards tips. Caudal fin membrane clear, vivid yellow at fork; rays light olive; rays lighter towards tips; melanophores small, dark, fading towards rear. Anal fin rays clear; membrane clear; dark spotting above origin; melanophores dark olive fading towards tips. Pectoral fin membranes clear; rays clear; first ray with few dark melanophores. Pelvic fin rays clear; membrane clear.

##### Preserved colouration.

(Fig. 7). Body and head white and silver with dark spotting sparse to densely packed towards caudal fin on midline and on dorsal surface. Scales on dorsal surface lightly pigmented. Ventral scale pigmentation as intense as dorsal surface. Dorsal surface of head lightly pigmented. Melanophores small, dark; clustered on rear of head, below orbit and on lips and snout; along midline, increasing in intensity to caudal fin; browner on dorsal surface above midline; forming a small, dark line above anal fin. Operculum and posterior edge of orbit with silver sheen. Membranes between fin rays clear. Pelvic fin clear membranes and rays. Dorsal, caudal and pectoral fin membranes clear; rays with melanophores small, dark, widely-spaced; rays pale grey.

##### Etymology.

‘*Brevianalis*’ alludes to the relatively short anal fin. ‘*Whitei*’ honours Mr A. G. White, who collected the type specimens in the Aapies River near Petronella and Hammanskraal.

##### Distribution.

Botswana, Zimbabwe, South Africa, Swaziland, Mocambique: Limpopo River, Incomati River, Pongolo River, St Lucia system, Mkhuze River.

##### Type locality.

Mkuzi River (perhaps within 40 km east or west of 27°35'S 32°00'E), South Africa.

##### Biology.

Pelagic species preferring close proximity to substrate and seeking out slacker areas such as backwater, eddies and pools below riffles. Occurs in shoals and prefers well-aerated, open water in flowing rivers (Skelton 2001), favouring the upper stratum (Engelbrecht and Mulder 1999). Feeds from water column on planktonic crustaceans and insects (e.g. midges and ants) (Skelton 2001). Caught at night with light. Breeding occurs in early summer (Skelton 2001). Found in dams where appears to propagate successfully with little predation and moves around in rivers according to seasonal flows. Appears to migrate up streams in spring to breed where it is found in tributaries.

##### Remarks.

The specimen (SAIAB 66270) used by Liao et al. (2012) to represent a DNA sequence of *Mesobola brevianalis* and is from the Usuthu River (Table 1), and does belong to that species (Fig. 3).

#### Engraulicypris gariepinus

Taxon classificationAnimaliaCypriniformesCyprinidae

Barnard, 1943 stat. rev.

Engraulicypris gariepinus Barnard, 1943. *Annals of the South African Museum***36**(2): 220. Syntypes: 2 unsexed (not located), “Orange River and Fish River” [SAM 18722–23] [lost].

##### Material examined.

SAIAB 193617, 6 unsexed, 2 cleared & stained: SL 43–44 mm. Namibia, Orange River, Noordower, 28°44'50"S 17°36'32"E, 21 October 2006, R. Bills; SAIAB 78822, 7 unsexed, 2 cleared & stained: SL 39–41 mm. Namibia, Orange River, Felix Unite Camp, 28°41'19"S 17°33'20"E, 20 October 2006, R. Bills; 2 unsexed, SAIAB 78805, 42–47 mm. Namibia, Orange River, Houms River Camp Site, 28°52'5"S 18°36'42"E, 18 October 2006, R. Bills; SAIAB 74232, 10 unsexed, 2 cleared & stained: SL 29–41 mm. South Africa, Orange River, Pella Drift lower site, 28°57'47"S 19°6'36"E, 28 January 2004, R. Bills & N. Jones.

##### Diagnosis.

Caudal fin membrane clear to pale orange towards midline; anal fin extending over three quarters of length of caudal peduncle; caudal peduncle short; operculum entirely (not partially) shiny; body midline silver (not black); iris dark to light grey (not white); head with tubercles along lower jaw and lower head in breeding males; snout rounded, with dense dark spotting on tip; pelvic fin melanophores absent.

##### Morphology.

(Figs 6–8; Table 6). Maximum SL 46 mm. Body elongated; somewhat fusiform; laterally compressed. Maximum body depth before pelvic fin. Pre-dorsal profile straight or slightly convex behind head. Head length 21% SL; with tubercles along lower jaw and lower head. Snout rounded; short; 32% of head length. Mouth terminal; slightly crescent-shaped with long anterior side; reaching anterior border of orbit. Nostrils large; level with dorsal margin of eye; separated from orbit by less than one orbit radius. Tubular anterior naris short; adjacent to open posterior naris. Eye lateral; visible from above and below (more prominent); diameter 32% of head length. First gill arch with 7+3 gill rakers on cerato- and epibranchial arms, respectively. Gill rakers long; pointed; widely-spaced. Pharyngeal bones in three rows. Pharyngeal teeth 4,3,2–2,3,4; robust and long; falcate.

**Table 6.** Morphometric measurements and meristic counts for *Engraulicypris gariepinus*.

Measurement	n	Max	Min	Mean	SD
SL	20	46.61	28.76	38.36	5.41
Head length (%SL)	20	23.78	18.25	21.18	1.79
Head depth (%HL)	20	93.35	59.71	77.75	7.75
Snout length (%HL)	20	40.37	23.10	31.77	4.69
Orbit diameter (%HL)	20	50.35	36.89	40.27	3.32
Postorbit length (%HL)	20	38.75	27.56	33.06	3.03
Inter-orbit length (%HL)	20	48.23	27.79	38.10	5.68
Predorsal length (%SL)	20	68.09	62.17	64.83	1.71
Prepelvic length (%SL)	20	55.17	45.07	49.03	2.32
Dorsal fin Length (%SL)	20	20.39	15.19	17.00	1.37
Pectoral fin length (%SL)	20	25.04	21.60	22.90	1.00
Pelvic fin length (%SL)	20	14.25	11.80	12.79	0.68
Anal fin length (%SL)	20	18.45	14.38	16.41	1.12
Body depth (%SL)	20	25.64	18.39	21.81	1.91
Body width (%SL)	20	13.27	9.39	11.11	1.19
Caudal peduncle length (%SL)	20	16.64	11.13	13.95	1.52
Caudal peduncle depth (%SL)	20	9.84	7.13	8.18	0.79
Meristics	n	Range			
Dorsal-fin rays	20	ii+7 (n = 3), ii+8 (n = 17)			
Anal-fin rays	20	iii+14 (n = 4), iii+15 (n = 9), iii+16 (n = 7)			
Pectoral-fin rays	20	i+9 (n = 10), i+10 (n = 8), i+11 (n = 2)			
Pelvic-fin rays	20	i+7 (n = 19), i+8 (n = 1)			
Lateral line scales	2	49 (n = 1), 51 (n = 1)			
Caudal peduncle scale	2	14 (n = 1), 16 (n = 1)			
Scale rows lat. line-dorsal	2	11 (n = 2)			
Scale rows lat. line-pelvic	2	2 (n = 2)			
Scale rows lat. line-anal	2	2 (n = 2)			
Total vertebrae	12	36 (n = 1), 38 (n = 3)			
Abdominal vertebrae	12	17 (n = 1), 18 (n = 1), 19 (n = 10)			
Caudal vertebrae	12	19 (n = 3), 20 (n = 8), 21 (n = 1)			
Rib pairs	12	13 (n = 3), 14 (n = 7), 15 (n = 2)			

Modal fin formulae in Table 6. Fins large in relation to body size. Dorsal fin closer to caudal fin than tip of snout; more or less above origin of anal fin; length 17% SL; posterior margin straight; rays soft; anterior-most branched fin ray longest. Pectoral fins largest; reaching ^1^/_2_ to ^3^/_4_ distance to base of pelvic fin; fin lacking lobe at base. Pelvic fins reaching ^2^/_3_ distance to base of anal fin; relatively small; pointed; fin lacking a basal lobe. Anal fin moderately long; extending over ^3^/_4_ length of caudal peduncle; last unbranched ray longest. Ano-genital opening at anterior of base of anal fin. Caudal peduncle short; half of length. Caudal fin forked; lobes pointed; upper lobe shorter.

Scales small to medium relative to body size; in regular rows; cycloid, slightly elongated; radially striate. Base of anal fin lacking sheath of enlarged, elongate scales. Lateral line present; complete; dipping drastically towards ventral at tip of pectoral fin; joins midline at posterior of caudal peduncle; scale count 49–51 (n = 2) along lateral line, 14–16 around caudal peduncle.

##### Live colouration.

(Fig. 6). Body without vertical bars or bands. Dorsum transparent pale brown with melanophores concentrated around dorsal fin; midline silver. Snout with dense dark spotting on tip. Operculum entirely metallic silver. Iris dark to light grey. Dorsal fin membrane clear; rays clear; melanophores fading towards tips. Caudal fin membrane clear to pale orange towards midline; rays dark grey, lighter towards tips; melanophores small, dark, fading towards rear. Anal fin rays clear; membrane clear; pale orange spotting above origin; melanophores few to absent. Pectoral fin membranes clear; rays clear; first ray few dark melanophores. Pelvic fin rays clear; membrane clear.

##### Preserved colouration.

(Fig. 7). Body and head orange with small dark brown spotting along dorsal surface, midline and above anal fin. Scales on dorsal surface lightly pigmented. Ventral scale pigmentation less intense than dorsal. Dorsal surface of head lightly pigmented. Melanophores small, dark; grouped on rear of head, below orbit, and on lips and snout; along midline, increasing in intensity to caudal fin; brownish on dorsal surface, darkening between origin of pectoral and dorsal fin; forming small dark line above anal fin. Membranes between fin rays clear. Pelvic fin clear membranes and rays.

##### Etymology.

‘*Gariepinus*’ refers to the Gariep, a San name for the Orange River that means ‘Great water’.

##### Distribution.

South Africa, Namibia: Lower Orange River system, Fish River (Barnard 1943).

##### Type locality.

Orange River and Fish River, Namibia (Barnard 1943).

##### Biology.

This shoaling fish favours open, shallow water, normally occurring in slack pools and particularly below riffles. Populations found in the lower Orange and Fish Rivers are limited by the Augrabies and Fish River Falls. They are thought to feed mainly on small autochthonous invertebrates (planktonic crustaceans or insects), and are caught in large numbers where they occur. They are restricted to turbid waters, which provide protection from visual predators (R. Bills, pers. obs.).

##### Remarks.

The two syntypes of *Engraulicypris gariepinus* Barnard, 1943 were originally stored in the South African Museum, but were moved to the Albany Museum, Grahamstown, South Africa (AMG 106 and 1009) (Eschmeyer 2014). The Albany Museum fish collection has now been moved to SAIAB and these specimens have not been traced (I.R. Bills, pers. obs.). There is no ‘exceptional need’ (ICZN, Articles 75.2 and 75.3) for a neotype, since there is only one species of *Mesobola* in the topotypical river system, and the species is sufficiently physically distinctive that even if another species was introduced, they would be easy to distinguish on the basis of published descriptions.

#### Engraulicypris howesi

Taxon classificationAnimaliaCypriniformesCyprinidae

Riddin, Bills & Villet sp. n.

http://zoobank.org/0476418B-6254-48D1-B336-69A8A4C56D33

##### Holotype.

SAIAB 201623, unsexed, SL 43 mm, “Olushandja Dam at channel outlet, Kunene River System, Namibia, 17°25'53"S 14°38'36"E, 16 April 2015, R. Bills, V. Bills & R. van Zeeventer, D-net”. In 70% ethanol [SAIAB]

##### Paratypes.

SAIAB 39012, 11 unsexed, SL 21–43 mm, 30 May 1992, C. Hay, N. James & P. Skelton; SAIAB 78759, 7 unsexed, SL 28–37 mm, Kunene River at Hippo Pool below Ruacana Waterfall, Namibia, 17°24'24"S 14°13'1"E, 21 August 2006, E. Swartz & Kramer; SAIAB 38961, 14 unsexed, SL 29–35 mm, 27, Kunene River, Namibia,17°38'33"S 14°21'67"E, 27 May 1992, C. Hay, N. James & P. Skelton, SAIAB 35340, 6 unsexed, 2 cleared & stained, Kunene River below Ruacana Falls, Namibia, 13 January 1991, B. van der Waal [SAIAB].

##### Diagnosis.

Anal fin extending over three quarters of length of caudal peduncle; caudal peduncle short; operculum entirely (not partially) shiny; body midline silver (not black); iris dark to light grey (not white); head with tubercles along lower jaw and lower head in breeding males; snout rounded; pelvic fin melanophores absent.

##### Morphology.

(Figs 6–8; Table 7). Maximum SL 43 mm. Body elongated; somewhat fusiform; laterally compressed. Maximum body depth midway along body. Pre-dorsal profile straight or slightly convex behind head. Head length 23% of SL; with tubercles along lower jaw and lower head. Snout rounded; short; 29% of head length. Mouth terminal; slightly crescent-shaped with long anterior side; reaching anterior border of orbit. Nostrils large; level with dorsal margin of eye; separated from orbit by less than one orbit radius. Tubular anterior naris short; adjacent to open posterior naris. Eye lateral; visible from above and below (more prominent); diameter 41% of head length. First gill arch with 8+3 gill rakers on cerato- and epibranchial arms, respectively. Gill rakers long; pointed; widely-spaced. Pharyngeal bones in four rows. Pharyngeal teeth 5,3,2,1–1,2,3,5; slender and long; falcate.

**Table 7.** Morphometric measurements and meristic counts for *Engraulicypris howesi*.

Measurement	N	Holotype	Max	Min	Mean	SD
SL	20	42.84	42.84	21.90	33.35	5.03
Head length (%SL)	20	21.62	25.04	21.43	22.76	1.11
Head depth (%HL)	20	64.25	74.52	56.81	65.62	5.61
Snout length (%HL)	20	25.27	34.30	21.22	28.53	3.95
Orbit diameter (%HL)	20	37.80	46.29	32.49	40.62	3.42
Postorbit length (%HL)	20	37.37	43.86	29.95	36.34	4.01
Inter-orbit length (%HL)	20	32.07	43.25	5.79	33.35	8.38
Predorsal length (%SL)	20	62.61	67.19	62.61	64.78	1.33
Prepelvic length (%SL)	20	46.27	51.11	40.37	47.48	2.57
Dorsal fin Length (%SL)	20	16.15	17.63	9.52	14.30	2.43
Pectoral fin length (%SL)	20	20.12	24.16	15.50	18.86	2.17
Pelvic fin length (%SL)	20	11.83	14.19	9.05	12.06	1.19
Anal fin length (%SL)	20	15.90	16.80	9.22	13.69	2.16
Body depth (%SL)	20	18.49	20.42	14.22	17.83	1.59
Body width (%SL)	20	10.04	10.96	5.82	9.36	1.46
Caudal peduncle length (%SL)	20	18.98	18.98	13.15	15.91	1.52
Caudal peduncle depth (%SL)	20	9.45	9.86	7.01	8.51	0.92
Meristics	n	Holotype	Range			
Dorsal-fin rays	20	ii+8	ii+6 (n = 2), ii+7 (n = 6), ii+7 (n = 12)			
Anal-fin rays	20	iii+13	iii+13 (n = 9), iii+14 (n = 6), iii+15 (n = 5)			
Pectoral-fin rays	20	i+10	i+8 (n = 2), i+9 (n = 17), i+10 (n = 1)			
Pelvic-fin rays	20	i+7	i+6 (n = 1), i+7 (n = 17), i+8 (n = 2)			
Lateral line scales	2	Unknown	51 (n = 1), 52 (n = 1)			
Caudal peduncle scale	2	Unknown	14 (n = 2)			
Scale rows lat. line-dorsal	2	Unknown	9 (n = 2)			
Scale rows lat. line-pelvic	2	Unknown	2 (n = 2)			
Scale rows lat. line-anal	2	Unknown	2 (n = 2)			
Total vertebrae	11	38	38 (n = 3), 39 (n = 7), 40 (n = 1)			
Abdominal vertebrae	11	19	19 (n = 10), 20 (n = 1)			
Caudal vertebrae	11	19	19 (n = 4), 20 (n = 7)			
Rib pairs	11	14	13 (n = 5), 14 (n = 6)			

Modal fin formulae in Table 7. Fins large in relation to body size. Dorsal fin closer to caudal fin than tip of snout; more or less above origin of anal fin; length 14% of SL; posterior margin straight; rays soft; anterior-most branched fin ray longest. Pectoral fins largest; reaching ^1^/_2_ to ^3^/_4_ distance to base of pelvic fin; fin lacking lobe at base. Pelvic fins reaching ^2^/_3_ distance to base of anal fin; relatively small; pointed; fin lacking a basal lobe. Anal fin moderately long; extending ^2^/_3_ length of caudal peduncle; last unbranched ray longest. Ano-genital opening at anterior of base of anal fin. Caudal peduncle moderately long; depth half of length. Caudal fin forked; lobes pointed; upper lobe shorter.

Scales small to medium relative to body size; in regular rows; cycloid; radially striate; rounded, slightly elongate. Base of anal fin lacking sheath of scales. Lateral line present; complete; dipping sharply towards ventral at tip of pectoral fin; joins midline at posterior of caudal peduncle; scale count 51–52 (n = 2) along lateral line, 14 around caudal peduncle.

##### Live colouration.

(Fig. 6). Body without vertical bars or bands. Dorsum transparent brown with melanophores concentrated around dorsal fin and caudal peduncle; midline silver. Snout darker dorsally. Operculum entirely metallic silver. Iris white to light grey. Dorsal fin membrane clear; rays clear with dark melanophores. Caudal fin membrane clear; rays dark brown to black, lighter towards edge; melanophores lighter towards tip. Anal fin rays clear; membrane clear; few dark spots above origin; melanophores absent. Pectoral fin membrane clear; rays clear; first ray with few dark melanophores. Pelvic fin rays clear; membrane clear.

##### Preserved colouration.

(Fig. 7). Body and head orange with small dark brown spots along dorsal surface, midline and above anal fin. Scales on dorsal surface lightly pigmented. Ventral scale pigmentation less intense than dorsal. Dorsal surface of head lightly pigmented. Melanophores small, dark; grouped on rear of head, below orbit, and on lips and snout; along midline, increasing in intensity to caudal fin; browner on dorsal surface, darkening between origin of pectoral and dorsal fin; forming small dark line above anal fin. Operculum with silver sheen. Side of body with silver sheen extending from pectoral fin to anal fin origin. Membranes between fin rays white to clear towards end. Pelvic fin clear membranes and rays. Dorsal, caudal and pectoral fin membranes white to clear; rays with small, widely-spaced, melanophores fading towards edges; rays pale brown to clear.

##### Etymology.

This species is named in honour of Gordon John Howes (1938-2013), whose studies of the osteology of the Danioninae (Howes 1980, 1984) laid the foundations of their modern classification. The epithet is a genitive noun.

##### Distribution.

Namibia, Angola: Cunene River system.

##### Type locality.

Olushandja Dam at channel outlet (17°25’53’’S 14°38’36’’E), Kunene River System, Namibia.

##### Biology.

Very little is known of the biology of this species. Individuals appear to favour turbid, rocky, river regions where they can gather in pockets of recirculating currents. The holotype and some paratypes were collected in the shallow, turbid Olushandja Dam in the Namibian upper reaches of the system. They feed on drifting invertebrate larvae and adults and plankton.

#### Engraulicypris ngalala

Taxon classificationAnimaliaCypriniformesCyprinidae

Riddin, Villet & Bills sp. n.

http://zoobank.org/5A3FD50F-25DF-49B8-86BD-EB911A238DFF

##### Holotype.

SAIAB 74087 A, GenBank KX788909, unsexed, SL 40 mm. “Lucheringo River, Singa Hunting Camp, Mozambique, 11°48'56"S 36°13'15"E, 25 August 2003, I.R. Bills, seine net”. In 70% ethanol [SAIAB].

##### Paratypes.

SAIAB 193064, 2 unsexed, SL 42-45 mm, collected with holotype; SAIAB 73944, 29 unsexed, 2 cleared & stained, SL 18–29 mm, Rovuma River below Chamba, Mozambique, 12°35'47"S 36°56'8"E, 19 August 2003, I.R. Bills; SAIAB 39269, 11 unsexed, 1 cleared and stained, SL 42–53 mm. Lake Chiuta at Mthubula Beach, Malawi, 14°78'33"S 35°83'33"E, 13 July 1992, P. Skelton & D. Tweddle [SAIAB].

##### Diagnosis.

Operculum shiny only on ventral posterior edge and small area at posterior edge of orbit (not entire area); body midline black (not silver); head with tubercles along lower jaw and lower head in breeding males; snout rounded (not pointed); iris white to light grey (not dark grey) with a few melanophores; pelvic fin melanophores present, dark and widely dispersed.

##### Morphology.

(Figs 6–8; Table 8). Maximum SL 51 mm. Body elongated; somewhat fusiform; laterally compressed. Maximum body depth midway along body. Pre-dorsal profile straight or slightly convex behind head. Head length 18% of SL; with tubercles along lower jaw and lower head. Snout rounded; short; 33% of head length. Mouth terminal; slightly crescent-shaped with long anterior side. Nostrils large; level with dorsal margin of eye; separated from orbit by less than one orbit radius. Tubular anterior naris short; adjacent to open posterior naris. Eye lateral; visible from above and below (more prominent); diameter 43% of head length. First gill arch with 13+3 gill rakers on cerato- and epibranchial arms, respectively. Gill rakers long; pointed; widely-spaced. Pharyngeal bones in four rows. Pharyngeal teeth 5,3,2,1–1,2,3,5; slender and long; falcate.

**Table 8.** Morphometric measurements and meristic counts for *Engraulicypris ngalala*.

Measurement	n	Holotype	Max	Min	Mean	SD
SL	20	40.03	50.46	19.37	43.95	2.90
Head length (%SL)	20	7.59	22.79	16.90	18.44	1.26
Head depth (%HL)	20	5.67	77.08	56.85	73.59	2.75
Snout length (%HL)	20	2.54	40.91	15.34	33.10	3.28
Orbit diameter (%HL)	20	3.47	46.01	30.50	43.01	2.67
Postorbit length (%HL)	20	2.54	38.22	22.50	33.18	3.24
Inter-orbit length (%HL)	20	2.68	43.41	21.26	39.13	2.46
Predorsal length (%SL)	20	25.35	68.23	62.27	64.10	1.82
Prepelvic length (%SL)	20	19.27	51.15	42.62	47.72	2.34
Dorsal fin Length (%SL)	20	5.43	19.27	11.89	14.04	1.81
Pectoral fin length (%SL)	20	8.88	23.15	19.29	21.84	0.98
Pelvic fin length (%SL)	20	5.01	17.66	10.57	13.20	1.53
Anal fin length (%SL)	20	6.31	17.52	12.66	14.57	0.97
Body depth (%SL)	20	7.46	20.68	13.89	18.37	0.99
Body width (%SL)	20	3.43	9.26	3.05	8.07	0.69
Caudal peduncle length (%SL)	20	5.98	18.31	12.82	15.89	1.32
Caudal peduncle depth (%SL)	20	3.24	10.13	5.33	8.46	0.64
Meristics	n	Holotype	Range			
Dorsal-fin rays	20	ii+7	ii+7 (n = 15), ii+8 (n = 5)			
Anal-fin rays	20	iii+14	iii+13 (n = 3), iii+14 (n = 6), 3+15 (n = 9), iii+16 (n = 2)			
Pectoral-fin rays	20	i+10	i+ 8 (n = 3), i+9 (n = 11), i+10 (n = 6)			
Pelvic-fin rays	20	i+7	i+6 (n = 2), i+7 (n = 17), i+8 (n = 1)			
Lateral line scales	2	Unknown	51 (n = 1), 52 (n = 1)			
Caudal peduncle scale	2	Unknown	14 (n = 1), 16 (n = 2)			
Scale rows lat. line-dorsal	2	Unknown	9 (n = 2)			
Scale rows lat. line-pelvic	2	Unknown	2 (n = 2)			
Scale rows lat. line-anal	2	Unknown	1 (n = 1), 2 (n = 1)			
Total vertebrae	14	38	38 (n = 2), 39 (n = 1)			
Abdominal vertebrae	14	19	19 (n = 12), 20 (n = 2)			
Caudal vertebrae	14	19	19 (n = 4), 20 (n = 10)			
Rib pairs	14	14	14 (n = 1), 15 (n = 13)			

Modal fin formulae in Table 8. Fins large in relation to body size. Dorsal fin closer to caudal fin than tip of snout; more or less above origin of anal fin; length 14% of SL; posterior margin straight; rays soft; anterior-most branched fin ray longest. Dorsal and anal fin point parallel. Pectoral fins largest; reaching ^1^/_2_ to ^3^/_4_ distance to base of pelvic fin; fin lacking lobe at base. Pelvic fins reaching ^2^/_3_ distance to base of anal fin; relatively small; pointed; fin lacking a basal lobe. Anal fin moderately long; extending ^2^/_3_ length of caudal peduncle; last unbranched ray longest. Ano-genital opening at anterior of base of anal fin. Caudal peduncle moderately long; depth half of length. Caudal fin forked; lobes slightly concave interior lobe into point; upper lobe shorter.

Scales small to medium relative to body size; in regular rows; cycloid; radially striate; rounded, slightly elongate. Base of anal fin lacking sheath of enlarged, elongate scales. Lateral line present; complete; dipping sharply towards ventral at tip of pectoral fin; joins midline at posterior of caudal peduncle; scale count 51–52 (n = 2) along lateral line, 14-16 (n = 3) around caudal peduncle.

##### Live colouration.

(Fig. 6). Body and head white ventrally with pale brown dorsal surface. Body midline black; colouration without vertical bars or bands. Dorsal surface with ubiquitous melanophores. Snout with dense dark spotting on tip. Operculum shiny only on ventral posterior edge and small area at posterior edge of orbit. Iris white to light grey with a few melanophores. Dorsal fin membrane clear; rays clear with dark melanophores. Caudal fin membrane clear to pale orange towards midline; rays dark brown to black, lighter towards edge; melanophores abundant and fading towards tips. Anal fin rays clear; membrane clear; pale orange spotting above origin; melanophores dark brown fading towards tips. Pectoral fin membranes clear; rays clear; first ray with abundant dark melanophores. Pelvic fin rays clear; membrane clear.

##### Preserved colouration.

(Fig. 7). Body and head pale yellow with dark brown spotting on dorsal surface and midline. Scales on dorsal surface lightly pigmented. Ventral scale pigmentation less intense than dorsal. Dorsal surface of head lightly pigmented. Melanophores small, dark; grouped on rear of head, below orbit, and on lips and snout; along midline, increasing in intensity to caudal fin; browner on dorsal surface, darkening between origin of pectoral and dorsal fin; forming small dark line above anal fin. Operculum and posterior base on orbit with silver sheen. Membranes between fin rays white to clear towards end. Pelvic fin clear membranes with melanophores on first ray. Dorsal, caudal and pectoral fin rays with melanophores small, widely-spaced, fading towards edges; pale brown to clear.

##### Etymology.

In the Cyao language spoken in the Niassa region of northern Mozambique, the name ‘*ngalala*’ denotes any, small, compressed, silvery fish, including *Mesobola* and species of *Brycinus* Valenciennes, 1850 and *Hemigrammopetersius* Pellegrin, 1926. The epithet is treated as a nominative singular noun in apposition.

##### Distribution.

Mozambique, Malawi: Rovuma River system and Lake Chiuta.

##### Type locality.

Lucheringo River below rapids at Singa hunting camp (11°48'56"S 36°13'15"E), Mozambique.

##### Biology.

This species is found in ecological conditions very similar to those characteristic of *Engraulicypris gariepinus* (Bills 2004). It favours big rivers, gathering in slack, turbid and shallow regions with sandy, rocky or muddy substrates. In Lake Chiuta specimens were caught in reed beds along the margins. The Lake Chiuta and Rovuma River stocks may differ ecologically because Lake Chiuta offers a lacustrine pelagic and benthic prey community (copepods, etc.) that is not found in the Rovuma River channel, where fish would predominantly have access to invertebrate drift.

## Supplementary Material

XML Treatment for Engraulicypris

XML Treatment for Engraulicypris brevianalis

XML Treatment for Engraulicypris gariepinus

XML Treatment for Engraulicypris howesi

XML Treatment for Engraulicypris ngalala
